# Successful live birth in a woman with resistant ovary syndrome following in vitro maturation of oocytes

**DOI:** 10.1186/s13048-016-0263-6

**Published:** 2016-09-06

**Authors:** Yu Li, Ping Pan, Ping Yuan, Qi Qiu, Dongzi Yang

**Affiliations:** Reproductive Medicine Centre, Department of Obstetrics and Gynecology, Sun Yat-Sen Memorial Hospital of Sun Yat-Sen University, 107 West Yan Jiang Road, Guangzhou, Guangdong 510120 China

**Keywords:** Resistant ovary syndrome, In vitro maturation of oocytes, Infertility

## Abstract

**Background:**

Resistant ovary syndrome (ROS) is a rare endocrine disorder characterized with hypergonadotrophic hypogonadism. Infertility is a common complaint of woman presenting with ROS, and little progress has been made in term of reproduction with the patient’s own gamete. So far only one case report of live birth has been reported after in vitro maturation (IVM) of oocytes in a patient suffering from ROS in 2013.

**Case presentation:**

A secondary infertile woman of 33 years-old was manifested with oligomenorrhea and markedly increased gonadotropin levels around postmenopausal range, but had normal antral follicle count, normal serum inhibin B and anti-Müllerian hormone levels. She had normal karyotype of 46,XX and normal thyroid function. There were no abnormal findings in some autoantibody assays and FSH receptor sequencing. After oral contraceptive pills combined with triptorelin depot were administered, her gonadotropin levels reduced but it showed no response to high doses of exogenous gonadotropins (hp-HMG 300IU/d for 15 days). Then endometrium was prepared with estradiol valerate and IVM from small antral follicles were performed. Five immature oocytes were retrieved. Twenty-four hours after IVM culture, 3 oocytes matured to metaphase II stage and were inseminated by intracytoplasmic sperm injection using her husband’s sperm. Two top-quality embryos were transferred and one embryo was cryopreserved. The patient got pregnant and delivered a healthy boy at term.

**Conclusion:**

IVM using their own oocytes could be an available treatment for infertile women with ROS.

## Background

Resistant ovary syndrome (ROS) is a rare endocrine disorder characterized with hypergonadotrophic hypogonadism, which was first described in 1969 [1]. Infertility is a common complaint of woman presenting with ROS at reproductive age, and indeed treatment is a great challenge for both clinicians and patients. Little progress has been made in term of reproduction with the patient’s own gamete. Several researchers have reported successful return of menstruation after ovarian biopsy, normalization of ovarian function and subsequent spontaneous pregnancy after hormone replacement therapy in very few cases [2, 3]. Patients with ROS usually have to accept egg donation because ovarian follicles were unresponsive to endogenous or exogenous FSH [4].

With the breakthrough in assisted reproductive technique, in vitro maturation (IVM) of oocytes has emerged as a reliable option in the management of infertility, and even been expanded in some uncommon situations [5]. To the best of our knowledge, so far only one case report of live birth has been reported after IVM of oocytes in a 29-years old patient suffering from ROS in 2013 [6]. The case we now presented here was the second one. However, we had ever tried to use IVM to treat a patient of ROS as early as in 2005, available oocytes and embryos were successfully obtained but regrettably the patient was not pregnant after embryo transfer. Here we shared this case report aiming to provide further evidence to support the effectiveness of IVM in ROS patients.

## Case presentation

### Patient history

In August 2014, a 33-year-old multiparous woman with an 8-years history of secondary infertility was referred to our IVF center. She had got married for 10 years and gave a normal child birth in 2005. After delivery she used intrauterine device (IUD) for contraception and IUD was released one year later. But she failed to conceive ever since. In 2008, hysterosalpingography showed bilateral salpingitis with bilateral hydrosalpinx and PPD tuberculin test was strong positive. Diagnosed doubtfully as pelvic tuberculosis in local hospital, then she received anti tuberculosis treatment for one year. In 2013, hysteroscopy revealed normal uterine cavity, and proliferative endometrium was confirmed by histopathology examination. Her menarche occurred at 16 years old and she had regular menses previously. Since 2008, she suffered from oligomenorrhea or amenorrhea with the cycle length of 40 to 90 days. She denied any endocrine or autoimmune disorders. She had a normal body mass index and did not present any clinical androgen excess signs. She was occasionally given oral contraceptive pills to induce menses. The study was approved by the ethics committee of Sun Yat-sen Memorial Hospital of Sun Yat-sen University. Informed consent has been obtained.

### Hormonal measurements and ultrasound scans

Initial hormone measurements and ultrasound scans were checked on day 3 of a spontaneous menstruation. Serum levels of FSH, LH, E_2_, prolactin and total testosterone were determined using an automated multianalysis system with chemiluminescence detection (UniCel Dxi 800 Acess, Beckman Coulter, USA). Serum AMH level was determined using ELISA (Ansh Labs, England). Serum inhibin B level was determined using a double antibody ELISA (Serotec). Ultrasound scans were performed using a 5.0- to 8.0-MHz multifrequency transvaginal probe (GE Voluson E8) by one single operator.

**Fig. 1:**
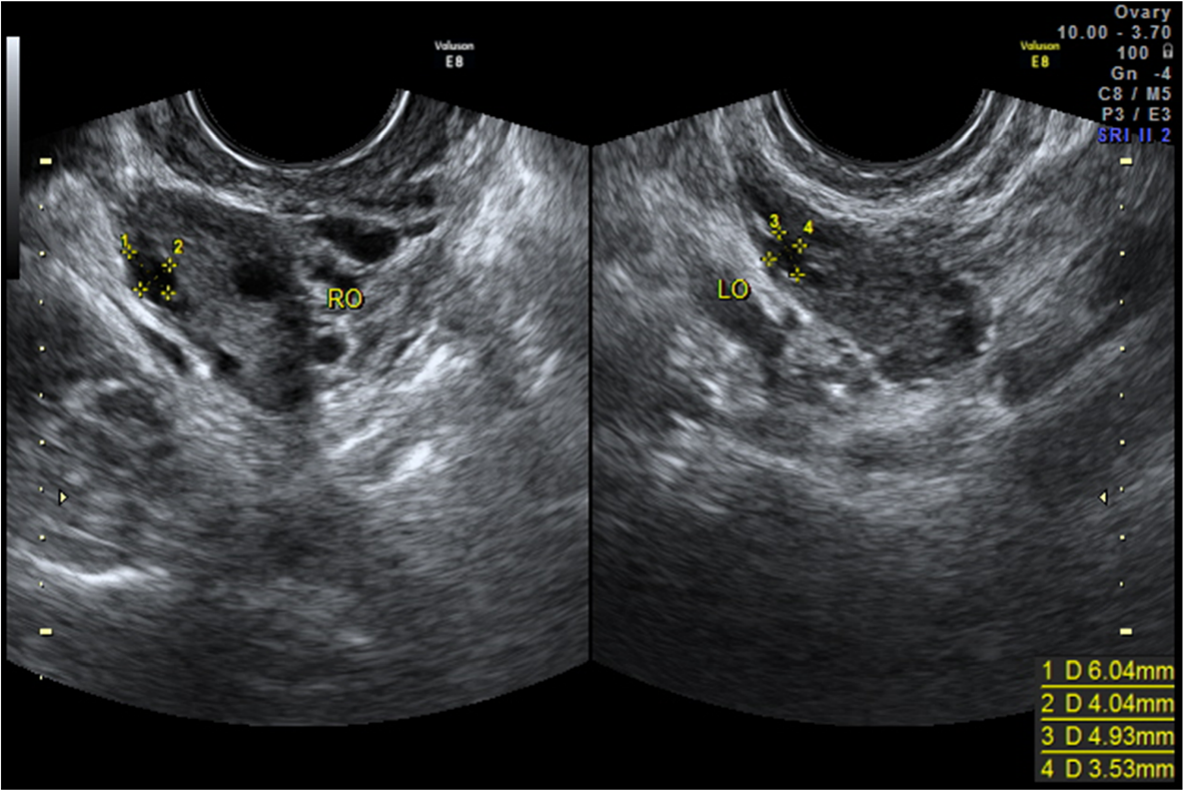
Transvaginal ultrasound scans of two ovaries with normal number of antral follicles, in line with normal AMH levels but in contrast with high serum FSH and LH

Initial hormonal profile demonstrated FSH and LH levels were around the menopausal range. While AMH and inhibin B levels were normal, and ultrasound scan showed normal size of uterus and ovaries with total 25 antral follicles of 2mm to 7mm in diameter. The patient was given by hormone replacement therapy (Femonton; Abbott Biologicals B.V., the Netherlands) to induce regular withdrawal bleeding for 3 months and during the time serial ultrasonic monitoring displayed no evidence of dominant follicular growth. Repeated hormonal evaluation on day 3 after withdrawal bleeding still showed high FSH and LH level. The hormonal and ultrasonographic profiles were described in details in Table 1 and Fig. 1.

**Table 1:** Hormonal and Ultrasonographic Profiles

	Initial measurement	Repeated measurements	Intra-assay CV (%)	Inter-assay CV (%)	Functional sensitivity
FSH (IU/l)	41.99	38.18 /42.41	4.30	/	0.20
LH (IU/l)	39.29	36.27 /46.21	5.40	/	0.20
E2 (pmol/l)	260.57	202.11/248.36	15.00	/	73.40
AMH (ng/ml)	12.27	/	≤10	≤15	0.06
Inhibin B (pg/ml)	40.59	/	≤10	≤15	10
Antral follicle count	25	20/24	/	/	/
T(nmol/l)	1.24	/	3.93	7.08	0.35
PRL (mIU/L)	188.68	/	1.42	/	5.30
TSH(mIU/L)	1.79	/	2.30	1.40	0.005
FT4(pmol/L)	16.72	/	2.23	4.00	1.30
FT3(pmol/L)	4.61	/	2.35	/	0.30
Anti-TPO(IU/mL)	<28.0	/	6.80	3.40	28.0
Anti-TG(IU/mL)	<15.0	/	6.30	7.00	25.0

*CV* coefficients of variation, *TSH* thyroid stimulating hormone, *T4* total thyroxine, *FT4* free thyroxine, *T3* total triiodothyronine, *FT3* free triiodothyronine, *Anti-TPO* thyroid peroxidase antibody, *Anti-TG* thyroglobulin antibody

### Conventional chromosome analysis and FSH receptor sequencing

**Fig. 2:**
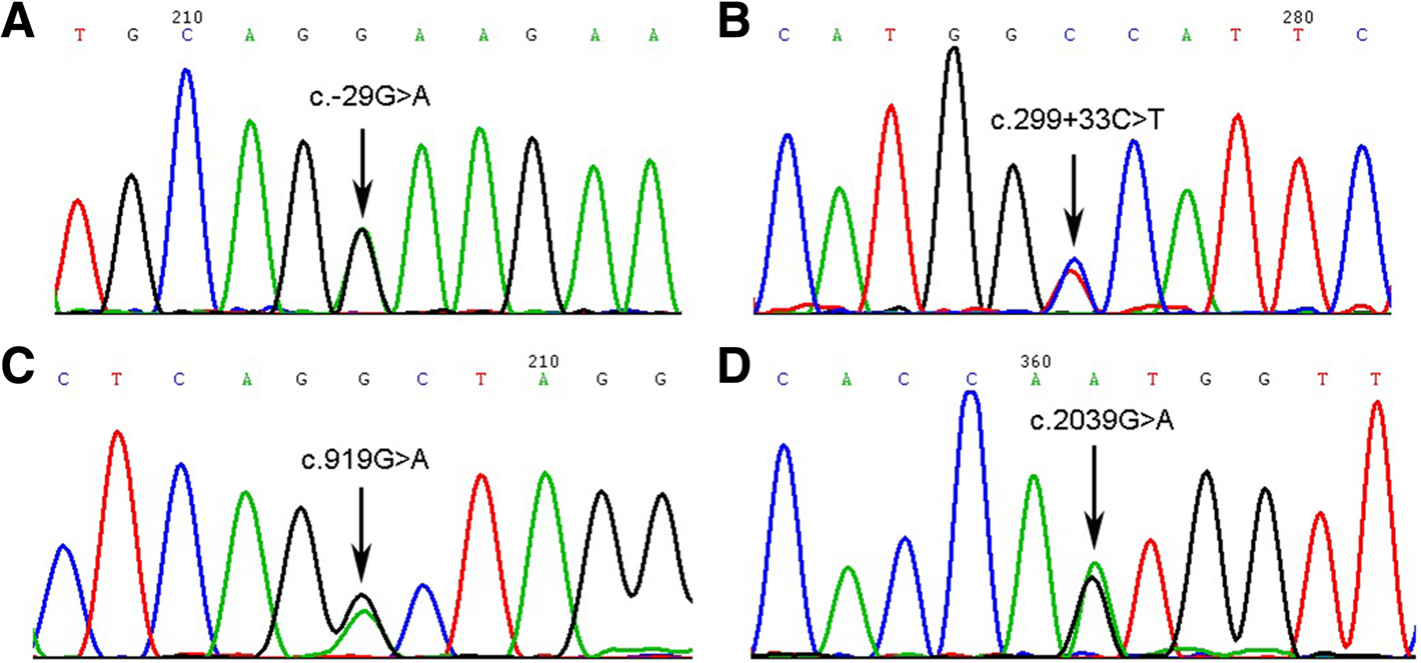
Partial sequencing results of genetic variations in the patient for the *FSHR* gene. **a** Forward sequences in the patient. The arrow indicates the heterozygous variation c.-29G > A of 5′-UTR. **b** Forward sequences in the patient. The arrow indicates the heterozygous variation c.299 + 33C > T of intron 3. **c** Forward sequences in the patient. The arrow indicates the heterozygous variation c.919G > A (p.Ala307Thr) of exon 10. **d** Forward sequences in the patient. The arrow indicates the heterozygous variation c.2039G > A (p.Ser680Asn) of exon 10

The patient’s karyotype showed normal 46,XX. Sanger sequencing identified 4 variants in the *FSHR* gene: c.-29G > A, c.299 + 33C > T, c.919G > A (p.Ala307Thr) and c.2039G > A (p.Ser680Asn). All of the 4 variants were identified benign variants by the use of SIFT (http://sift.jcvi.org/) and PolyPhen-2 (http://genetics.bwh.harvard.edu/pph2/) and searching the databases, the Human Gene Mutation database (http://www.hgmd.org/), the dbSNP (http://www.ncbi.nlm.nih.gov/snp). The details were shown in Fig. 2.

### Thyroid function and some autoantibody assays

Serum levels of thyroid function including thyroid stimulating hormone, total thyroxin, free thyroxin, total triiodothyronine, free triiodothyronine, thyroid peroxidase antibody and thyroglobulin antibody were determined using chemiluminescence detection (Siemens Healthcare Diagnostics Inc, USA). Serum antiovarian antibody was determined using ELISA (Shenzhen anquan Biotech Co., Ltd, China). Serum antizona pellucida antibody was determined using ELISA (Weifang kanghua Biotech Co., Ltd, China). There were no abnormal findings in thyroid function and these autoantibodies.

### Ovarian stimulation and IVM procedure

Twenty-one consecutive days of oral contraceptive pills (Marvelon; Organon Pharmaceutical Co Ltd, The Netherlands) were prescribed to the patient. On the 15th day of oral contraceptive pills, serum hormone concentrations dropped down: FSH 3.03 IU/L, LH 3.88 IU/L and E_2_ 73.40pmol/L, then a single dose of 1.25 mg triptorelin depot (Decapeptyl 3.75mg; Ferring Pharmaceuticals Ltd, The Netherlands) was administrated for pituitary down-regulation. After 14 days of triptorelin injection, which was on the sixth day of withdrawal bleeding, endogenous gonadotropin dropped to FSH 1.35 IU/L, LH 3.22 IU/L and E_2_ 104.14pmol/L. Then we started ovarian stimulation. Firstly, 100IU/d recombinant FSH beta (Puregon; Organon Pharmaceutical Co Ltd, The Netherlands) combined with 75IU/d highly purified menotrophin (Hp-hMG) (Menopur; Ferring Pharmaceuticals Ltd, The Netherlands) were administrated for 4 days. Ultrasound scan showed that no follicle developed larger than 5 mm in diameter and the thickness of endometrium was less than 4mm, as well serum E_2_ level was still low, 91.75 pmol/l. Then gonadotropin was changed to 300IU/d Hp-hMG for another 11 days, but it still showed that only small antral follicles, thin endometrium and unchanged serum E_2_ level. Considering the patient was unresponsive to endogenous and exogenous gonadotropin, we decided to try IVM procedures after a fully consultation with the couple. On following 7 days, 4mg/d estradiol valerate (Progynova; Bayer Schering Pharma, France) was given orally for endometrial preparation. Finally after 15-days ovarian stimulation and 7-days oral estradiol valerate, endometrial thickness reached to 8.0 mm and small follicles were still 2 to 5 mm in diameter. Then 10 000 IU human chorionic gonadotropin (hCG) (Lizhu Pharmaceutical Co, China) was administrated. About 36 h after hCG administration. 5 immature oocytes were retrieved by ultrasound guidance with 17G single-lumen aspiration needle (Cook; William Cook Australia Pty Ltd, Australia) under a reduced aspiration pressure of 10.7 kPa.

### IVM procedure

IVM medium (SAGE In-Vitro Fertilization, Inc. USA) at the concentration of 0.075IU/mL was prepared by 10 ml stock solution added with 100 ul Hp-hMG(Menopur; Ferring Pharmaceuticals Ltd, The Netherlands). Oocyte-cumulus complexes were matured in 1 ml IVM medium for 24 h, afterwards oocytes were denuded of cumulus cells and checked for maturity. Three out of five oocytes reached metaphase II stage and were inseminated by intracytoplasmic sperm injection (ICSI) using the partner’s spermatozoa, whereas two oocytes were still immature on germinal vesicle (GV) stage. After ICSI, three oocytes were normally fertilized and embryo evaluation and selection was performed 68 h after ICSI.

### Embryo transfer and follow-up

On day three after ICSI, three embryos were obtained and two top quality embryos with eight cells and <5 % fragmentation were transferred into the uterus, while one embryo was cryopreserved. Luteal support with 40 mg/day natural progesterone in oil (Lizhu Pharmaceutical Co, China) and oral administration of 20 mg/day dydrogesterone (Abbott Biologicals B.V., the Netherlands) were started on the day of ICSI, in combination with continuous estradiol valerate treatment. Fourteen days after embryo transfer, serum ß-hCG was positive and luteal support continued until 10 gestational-weeks. The pregnancy evolved without complications and she uneventfully delivered a healthy boy, 3200 g, at term.

## Discussions

Resistant ovary syndrome, previously known as Savage Syndrome, is a rare disease of unknown etiology. So far there are only a few reports described in the literature. The manifestations of ROS involve primary or secondary amenorrhea, normal secondary sexual characters, an age-compatible number of small antral follicles, normal chromosome, elevated gonadotropin levels of menopausal range, and unresponsiveness to gonadotropin stimulation. ROS might be misdiagnosed as primary ovarian insufficiency (POI). The main difference between ROS and POI is the presence of a normal amount of antral follicles in ROS, while few or no follicles are found in POI. In the past ovarian biopsy has been required to clarify a diagnosis of ROS, but nowadays many non-invasive approaches are helpful in proving the presence of antral follicles. It is well known that antral follicles as small as 2 mm in diameter can be visualized by high-resolution transvaginal ultrasonography [7]. Furthermore, hormone biomarkers such as inhibin B and AMH have been suggested to have a diagnostic role in women with ROS [4, 7], which are secreted by granulosa cells of growing follicles and closely related to ovarian reserve. In the report of Arici et al, Inhibin B has a diagnostic role in two women with ROS who manifested by secondary amenorrhea, high gonadotropin level but normal inhibin B level and normal primordial follicles after ovarian biopsy, while AMH level was not measured [4]. However, in the report of Grynberg et al, the case with ROS showed very low inhibin B level but normal AMH level [6]. The case in this report showed normal both inhibin B and AMH level. The conflicting results could be explained by the existence of different sizes of antral follicles in the ovary among different women with ROS. The intrafollicular concentrations of AMH become progressively lower with increasing follicle diameters but concentrations of inhibin B increased with increasing follicle diameter in human small antral follicles [8]. Thereafter AMH could be a better biomarker than inhibin B for discriminating ROS and POI, due to it correlates with ovarian primordial follicle number even after adjustment for chronological age [9]. According to the clinical manifestations and biomarkers, the patient in this report should be diagnosed as ROS.

ROS has long been an enigma. Mueller et al [3] reported there was no indication for any autoimmune disease in a patient with ROS and tests for antinuclear antibodies, antiphospholipid antibodies, lupus anticoagulant and anticardiolipin antibodies were all negative. Arici et al [4] also found no autoantibodies against thyroid, adrenals, or ovaries in two women with ROS. Grynberg et al [6] conducted more detailed genetic and autoimmune explorations in a 29-year-old patient, including karyotype, sequencing of FSH and LH receptor genes, analysis of GDF9, FOXL2, BMP15 genes and FRAXA mutation, as well as adrenal cortex autoantibodies, steroid cell autoantibodies, serum 21-hydroxylase, 17-hydroxylase and P450 side-chain cleavage enzyme autoantibodies, yet revealed no abnormality. Similarly, our case showed no history of autoimmune diseases, normal karyotype, no deleterious mutations in FSHR gene, and some negative autoantibodies. Gonadotropin receptor or hormone mutations were rarely found in sporadic cases of gonadotropin resistance [10, 11]. While ROS showed few links with genetics, since family history, chromosome and gonadotropin receptor as well as post-receptor defects analysis invariably proved to be normal [6, 12, 13]. Thereafter, gonadotropin resistance in ROS women might be due to defects in interactions of FSH and its receptor, mostly secondary to autoimmune activities [14, 15]. Some reports demonstrated that antigonadotropin antibodies were found in women with gonadotropin resistance [13, 16, 17]. ROS could be another special condition of reproductive autoimmune failure that has been described elsewhere [18, 19]. The patient in this report had a medical history of pelvic tuberculosis and oligomenorrhea or amenorrhea occurred after anti-tuberculosis therapy. While mycobacterial tuberculosis infections are known to induce the development of autoantibodies and even autoimmune diseases [20, 21]. In a recent study of ROS case, it demonstrated that IgG/IgM-antibodies directed against some forms of gonadotropins were found in serum and respective antibodies in this serum only react with some but not all forms of FSH [13]. And the expressed fashion of FSHR, LHR as well as estrogen receptor beta and progesterone receptor A induced by exogenous gonadotropins was not different in granulosa cells from ROS patient and from control patients [13]. But the ROS case in this report showed normal response to ovarian stimulation with daily 75IU recombinant beta and 225IU hp-HMG for 14 days after hormone replacement and GnRH agonist treatment. 11 mature oocytes were obtained and a successful live birth after embryo transfer [13]. Several common autoantibodies associated with infertility were not detected in our ROS case but it is limited that antigonadotropin antibodies have not been tested. It need be further investigated whether the possible mechanism of autoimmunity can explain gonadotropin resistance in all ROS patients, and the titers or types of antigonadotropin antibodies are related to that ovarian response has or not and which kind of exogenous gonadotropin should be effective.

Infertility is a difficult issue for ROS patients at reproductive age, and the chances of fertility with their own oocytes are unpredictable and most likely poor. Attempts to stimulate follicle development with high doses of gonadotropins to override the resistant state seem to be invalid [3, 4, 6]. Indeed we tried to induce follicle growth with 300IU daily hp-HMG for 15 days, but no response. Taking into consideration the first pregnancy and live birth after IVM in ROS case was reported in 2013 [6], we tried the same method. Three out of five oocytes were matured in vitro, and the transfer of two top-quality embryos resulted in a successful live birth. These two successful cases with ROS by IVM also confirm that granulosa cells can response to exogenous gonadotropin in vitro and IVM overcomes the state of gonadotropin resistance in ROS patients. Finally, no choice but IVF using donor oocytes was the only option for ROS in case of IVM and all other treatment failures [4], undoubtedly the reproductive prognosis was acceptable in the donor oocyte program.

## Conclusion

The ROS patient can obtain available oocytes/embryos and live birth by IVM even after she shows no response to ovarian stimulation with large dose of exogenous gonadotropin. IVM should be an option available for infertile women afflicted with resistant ovary syndrome.

## Abbreviations

AMH: Anti-Müllerian hormone
E_2_: Estradiol
GV: Germinal vesicle
hCG: Human chorionic gonadotropin
ICSI: Intracytoplasmic sperm injection
IUD: Intrauterine device
IVM: In vitro maturation
PCR: Polymerase chain reaction
POI: Primary ovarian insufficiency
PRL: Prolactin
ROS: Resistant ovary syndrome
T: Testosterone
